# Incorporating Oxygen-Enhanced MRI into Multi-Parametric Assessment of Human Prostate Cancer

**DOI:** 10.3390/diagnostics7030048

**Published:** 2017-08-24

**Authors:** Heling Zhou, Rami R. Hallac, Qing Yuan, Yao Ding, Zhongwei Zhang, Xian-Jin Xie, Franto Francis, Claus G. Roehrborn, R. Douglas Sims, Daniel N. Costa, Ganesh V. Raj, Ralph P. Mason

**Affiliations:** 1 Department of Radiology, University of Texas Southwestern Medical Center, Dallas, TX 75390, USA; heling.zhou@utsouthwestern.edu (H.Z.); Rami.Hallac@childrens.com (R.R.H.); Qing.Yuan@UTSouthwestern.edu (Q.Y.); YDing1@mdanderson.org (Y.D.); sljzzw@gmail.com (Z.Z.); chosims@sbcglobal.net (R.D.S.); Daniel.Costa@UTSouthwestern.edu (D.N.C.); 2 Analytical Imaging and Modeling Center, Children’s Medical Center, Dallas, TX 75235, USA; 3 Department of Radiation Oncology, University of Texas MD Anderson Cancer Center, Houston, TX 77030, USA (Present address); 4 Department of Clinical Sciences, University of Texas Southwestern Medical Center, Dallas, TX 75390, USA; Xian-Jin.Xie@UTSouthwestern.edu; 5 Department of Pathology, University of Texas Southwestern Medical Center, Dallas, TX 75390, USA; Franto.Francis@UTSouthwestern.edu; 6 Department of Urology, University of Texas Southwestern Medical Center, Dallas, TX 75390, USA; Claus.Roehrborn@UTSouthwestern.edu (C.G.R.); Ganesh.Raj@UTSouthwestern.edu (G.V.R.)

**Keywords:** prostate cancer, multi-parametric MRI, Gleason score, oxygen

## Abstract

Hypoxia is associated with prostate tumor aggressiveness, local recurrence, and biochemical failure. Magnetic resonance imaging (MRI) offers insight into tumor pathophysiology and recent reports have related transverse relaxation rate (R_2_*) and longitudinal relaxation rate (R_1_) measurements to tumor hypoxia. We have investigated the inclusion of oxygen-enhanced MRI for multi-parametric evaluation of tumor malignancy. Multi-parametric MRI sequences at 3 Tesla were evaluated in 10 patients to investigate hypoxia in prostate cancer prior to radical prostatectomy. Blood oxygen level dependent (BOLD), tissue oxygen level dependent (TOLD), dynamic contrast enhanced (DCE), and diffusion weighted imaging MRI were intercorrelated and compared with the Gleason score. The apparent diffusion coefficient (ADC) was significantly lower in tumor than normal prostate. Baseline R_2_* (BOLD-contrast) was significantly higher in tumor than normal prostate. Upon the oxygen breathing challenge, R_2_* decreased significantly in the tumor tissue, suggesting improved vascular oxygenation, however changes in R_1_ were minimal. R_2_* of contralateral normal prostate decreased in most cases upon oxygen challenge, although the differences were not significant. Moderate correlation was found between ADC and Gleason score. ADC and R_2_* were correlated and trends were found between Gleason score and R_2_*, as well as maximum-intensity-projection and area-under-the-curve calculated from DCE. Tumor ADC and R_2_* have been associated with tumor hypoxia, and thus the correlations are of particular interest. A multi-parametric approach including oxygen-enhanced MRI is feasible and promises further insights into the pathophysiological information of tumor microenvironment.

## 1. Introduction

Prostate cancer (CaP) is the most frequently diagnosed non-skin malignancy in men. The diagnosis of CaP requires invasive transrectal ultrasonography (US)-guided systematic prostate biopsies in patients with an elevated serum prostate-specific antigen (PSA) and/or abnormal digital rectal examination. If CaP is identified in the biopsies, then they are graded with the Gleason scoring system, which correlates with the CaP prognosis [1]. Prostate biopsy generally samples a small portion of the prostate, and may miss the cancer altogether, especially those in the anterior portion of the prostate. 

Magnetic resonance imaging (MRI) is an established modality for prostate evaluation, due to its high soft tissue contrast. In addition to providing high resolution imaging of the prostate gland, MRI can provide complementary functional tissue information, such as assessment of vascular permeability and perfusion. Several MRI techniques have been used to identify potential cancer areas within the prostate and certain MRI features have been shown to be associated with higher Gleason scores [2,3,4,5]. Multiparametric MRI is currently being used in our and other institutions for MRI-Transrectal Ultrasonography (TRUS) fusion biopsies [2,5]. Other MRI techniques like Diffusion Weighted MRI (DWI) explore the Brownian motion of water in tissue, and provide evaluation of tissue cellular density and membrane integrity, reported as apparent diffusion coefficient (ADC) values. ADC values are reported to correlate with aggressiveness of CaP [6,7], and may serve as an imaging biomarker to detect prostate tumor recurrence. Dynamic contrast enhanced (DCE) MRI evaluates vascular permeability and perfusion of the gadolinium contrast agent, with both semi-quantitative and quantitative parameters showing correlation with tumor severity and grade [8,9].

Increasing evidence suggests that hypoxia is associated with tumor aggressiveness, local recurrence and biochemical failure [10]. Past studies have examined prostate tumor hypoxia using needle electrodes or microarray expression analysis of biopsy specimens [11,12,13]. MR imaging approaches offer insight into tumor pathophysiology, and recent reports have related transverse relaxation rate (R_2_*) measurements to tumor hypoxia [12,14,15]. Particularly, blood oxygen level dependent (BOLD) MRI based on T_2_*-weighted (T_2_*w) contrast induced by paramagnetic deoxyhemoglobin concentration has shown promise in assessing CaP oxygenation in both patient [12,14,16] and animal studies [17,18,19]. We are only aware of three previous studies examining MRI response to hyperoxic gas breathing challenge in patients with CaP, each of which examined BOLD response to carbogen (5 and 2% CO_2_, respectively in oxygen [14,16,20]): we have now examined a pure oxygen breathing challenge, since this is expected to be less stressful. BOLD is sensitive to vascular oxygenation, but R_1_-based methods may more directly reflect tissue oxygenation, often referred to as tissue oxygen level dependent (TOLD) [21,22,23]. TOLD contrast comes from the T_1_ shortening effect of the paramagnetic oxygen molecule. Notably, hypoxia is recognized to play an important role in radiation resistance. Indeed, it has been reported that the response of Dunning prostate R3327-AT1 tumors to single high dose radiation (equivalent or stereotactic ablation radiotherapy: SABR) was related to TOLD, but not BOLD responses to hyperoxic gas breathing [24]. A recent study comparing the prognostic value of R_1_ and R_2_* responses to irradiation found correlation for BOLD response to carbogen breathing challenge in subcutaneous 9L gliomas in rats, but not rhabdomyosarcomas [25]. BOLD MRI response to oxygen breathing challenge has been related to radiation response in GH3 prolactinomas [26]. Correlations were also found with a split dose irradiation paradigm of R3327-AT1 tumors, with respect to R_2_* response to oxygen breathing challenges before the first dose [27], and with respect to the change in R_1_ response before the first and second doses [28], though trends were observed rather than tumor stratification. Intriguingly, tumor growth delay following single dose irradiation in Dunning Prostate R3327-AT1 tumors was related to the extravascular-extracellular volume fraction (VE), particularly in those tumors which did not benefit from oxygen breathing challenge [29], emphasizing the potential importance of exploring multi parametric imaging. TOLD has been applied to patients with oxygen breathing challenge [22,30], but does not appear to have been tested with respect to CaP in humans previously, and thus we have incorporated it into our present study.

In this pilot study, multi-parametric ^1^H MRI sequences, including BOLD, TOLD, DCE, and DWI have been evaluated in patients with biopsy-proven CaP prior to definitive surgical management with a radical prostatectomy. MRI findings have been correlated with the pathologic Gleason score in the prostatectomy specimens.

## 2. Materials and Methods

### 2.1. Magnetic Resonance Imaging (MRI) Study

This study was approved by the Institutional Review Board (IRB) and complied with the Health Insurance Portability and Accountability Act (IRB 1083 44000; 25 May 2010 and STU 012011-094; 5 May 2011). From April 2011 to November 2011, 10 consecutive men with biopsy confirmed CaP (mean age 59 years, mean prostate-specific antigen (PSA) level 6.9 ng/mL and Gleason score ranging from 6 to 9) signed IRB-informed consent and underwent multi-parametric MRI, as part of their preoperative workup. All ten patients had tumors in the peripheral zone. The post-surgery Gleason score, pathologic stage (pStage) and tumor size (longest diameter by pathology) of the 10 subjects are listed in Table 1. Images were acquired using six-element SENSitivity Encoding (SENSE) cardiac and endorectal receive coils on a 3 Tesla (T) scanner (Achieva, Philips Medical Systems, Cleveland, OH, USA). Anatomical high resolution T_2_-weighted (T_2_w) images were acquired in the transaxial plane using Turbo Spin Echo (TSE): TR/TE = 8869/120 ms, echo train length = 26, field of view (FOV) = 140 mm, reconstruction matrix size = 512 × 512, slice thickness = 3 mm. Additional non-standard MRI were acquired prospectively. R_1_ maps were acquired during baseline air breathing using T_1_ fast field echo (FFE) with variable flip angles (five flip angles from 2° to 14°), FOV = 240–260 mm, in-plane resolution 0.94 mm, slice thickness = 3 mm. Dynamic R_2_* maps (BOLD) were acquired using a multi-echo gradient echo sequence (T_1_-FFE; TR = 65 ms, 16 TE ranging from 1.7 to 39.2 ms, flip angle = 30°), FOV = 300 mm, in-plane resolution 0.94 mm, slice thickness = 6 mm. During the dynamic BOLD acquisition (0.5 min/dynamic), subjects breathed room air for three minutes to provide baseline data, then 100% oxygen (15 L/min) was delivered through a face mask (CareFusion, Chāteaubriant, France) for seven minutes to ensure stability in blood oxygenation.

R_1_ maps were acquired again during O_2_ breathing immediately after the dynamic R_2_* maps. Arterial oxygen saturation (SaO_2_) and heart rate were monitored throughout the exam using a pulse oximeter (In vivo 4500 MRI, In vivo Research Inc., Orlando, FL, USA). After turning off the oxygen, DWI was acquired with air-breathing using a single-shot spin-echo echo-planar sequence with b values of 0, 500, 1000 s/mm^2^, and TR/TE = 6228/70 ms. DCE was acquired using the T_1_ FFE sequence (TR = 4.5 ms, TE = 2.3 ms, flip angle 15°) with a bolus injection of 0.1 mmol/kg gadopentetate dimeglumine (Magnevist^®^; Bayer HealthCare, Wayne, NJ, USA) using a power injector at a rate of 2 mL/s and a 20 mL saline flush. Twenty-five dynamic phases were acquired with a temporal resolution of 12 s. 

### 2.2. Pathologic Analyses

Following radical prostatectomy, a visual map of the whole prostate was re-created from corresponding H&E histologic sections, to anatomically correspond to the in situ prostate. Areas of tumor were marked on the H&E slides and mapped onto a prostate map (with differential color assignment based on tumor Gleason grade), to provide reference for the MR images. These maps were created by an expert prostate pathologist (Franto Francis), without any prior knowledge of the MRI findings.

### 2.3. MRI Data Analysis

Image analysis was performed using custom programs written in MATLAB (MathWorks Inc., Natick, MA, USA). Regions of interest (ROIs) of lesions and contralateral normal prostate tissue were outlined by a radiologist based on T_2_-weighted images and pathology. Three patients had two lesions on the MRI and the index lesion was selected for analysis [31,32]. The lesion on the MRI was assigned the corresponding Gleason score at the same location on the pathologic map.

R_2_* maps during air and oxygen breathing were generated by fitting the multi-echo T_2_*w image intensity to TE from the multi-echo gradient echo sequence, as a monoexponential function on a voxel-by-voxel basis. ΔR_2_* was defined as average R_2_*(oxygen) − average R_2_*(air). A 3 × 3 2D Gaussian filter was applied to smooth the data. R_1_ maps were obtained through curve fitting with multiple flip angles:

(1) SI=M0×sinα×1−e−TRT11−cosα×e−TRT1

where SI is signal intensity, M0 is magnetization, and α is flip angle.

ADC maps were generated from the DW images automatically on the scanner. Histograms of voxel-by-voxel ADC values within each tumor ROI were calculated with a bin size 50 × 10^−6^ mm^2^/s.

Signal intensity time curves from the DCE scan were normalized to baseline, and time-to-maximum (TTM) enhancement, contrast uptake maximum-intensity-projection (MIP; maximum intensity reached), uptake slope, and area-under-the-curve (AUC) were extracted for ROIs and on a voxel-by-voxel basis.

### 2.4. Statistical Analysis

Although this was largely a feasibility/pilot trial, statistical analyses were applied. Student’s *t*-tests were performed to examine MR parameters of tumor and contralateral normal prostate (*p* < 0.05 was considered statistically significant). Pearson correlation was performed between MR parameters, and Spearman correlation was performed when correlating with Gleason score. Principal Component Analysis was performed on seven MR parameters (R_2_* air, ΔR_2_*, ADC, MIP, TTM, slope, and AUC) using Statistical Analysis System software (SAS Institute Inc., Cary, NC, USA). First and second Principal Components were obtained and correlated with Gleason score.

## 3. Results

**Figure 1:**
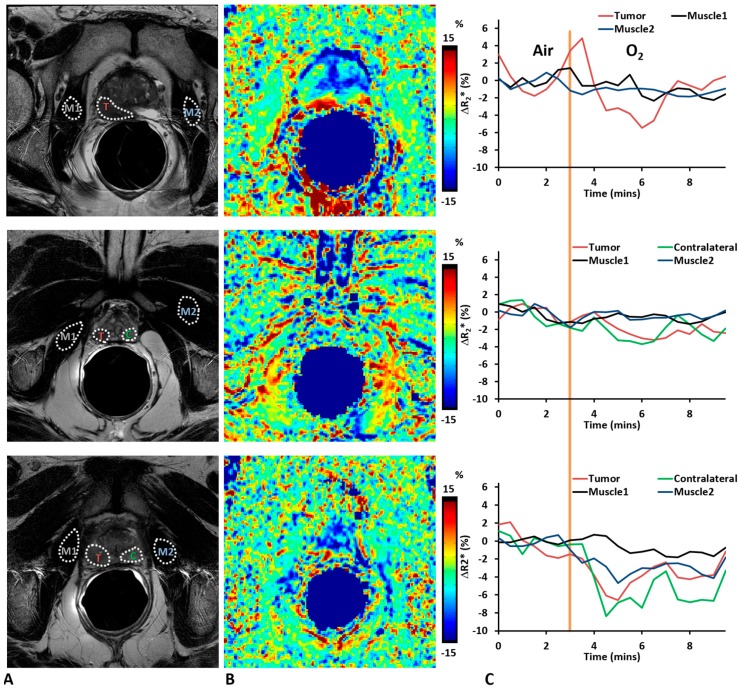
Oxygen-enhanced magnetic resonance imaging (MRI) of human prostate cancer. (**A**) T_2_w images showed hypointense tumor regions (T); (**B**) %ΔR_2_* maps (changes are normalized to baseline transverse relaxation rate (R_2_*) maps) for three representative tumors of Patients #3 (top), #5 (middle), and #6 (bottom) with Gleason scores 9, 7, and 8, respectively; (**C**) Traces of four regions of interest (ROIs, outlined in (**A**) with white dotted lines) showing dynamic changes of R_2_* in response to breathing oxygen (yellow line indicates start of oxygen breathing challenge). Tumor and normal prostate showed larger changes with oxygen breathing challenge compared to reference muscles. Labels in Figure 1A: Tumor (T), Contralateral normal prostate (C), Reference Muscle 1 and 2 (M1 and M2). Obturator internus or obturator externus muscles are selected as reference muscle. Note that in patient #3, no reliable normal prostate in the peripheral zone could be obtained due to susceptibility artefact in R_2_* maps, and the ROI of Contralateral normal prostate was omitted in this case.

The MRI studies were well tolerated, and high quality images were obtained from all 10 patients. Pulse oximetry showed significant increase in oxygen saturation in response to oxygen challenge (mean ± SD; 96.8 ± 1.3% with air to 99.3 ± 0.6% with 100% oxygen; *p* < 0.05), while the pulse rate during oxygen breathing (63.6 ± 12.6 beats/min) was not significantly different from baseline air (62.9 ± 12.1 beats/min). R_2_* measurements were obtained in tumor, contralateral normal prostate, and reference muscles, with the exception of one case (patient 3), where no reliable normal prostate in the peripheral zone could be obtained due to susceptibility artefact. Both tumor and normal prostate showed heterogeneity in baseline R_2_* and a range of BOLD signal responses to oxygen breathing challenge (Figure 1). The BOLD responses of reference muscles (obturator internus or obturator externus muscles) were smaller than those in tumor (paired Student’s *t*-test; not significant, *p* = 0.06) and contralateral normal prostate (paired Student’s *t*-test; not significant, *p* = 0.29). R_2_* of tumor decreased significantly (paired Student’s *t*-test; *p* < 0.01, *n* = 10) upon oxygen challenge (from 46.05 ± 20.84 s^−1^ to 44.41 ± 20.83 s^−1^), indicating improved oxygenation. R_2_* of contralateral normal prostate also decreased in seven out of nine cases upon oxygen challenge, although the differences were not significant (paired Student’s *t*-test; *p* = 0.13, *n* = 9; from 41.91 ± 17.71 s^−1^ to 40.94 ± 18.19 s^−1^). 

**Figure 2:**
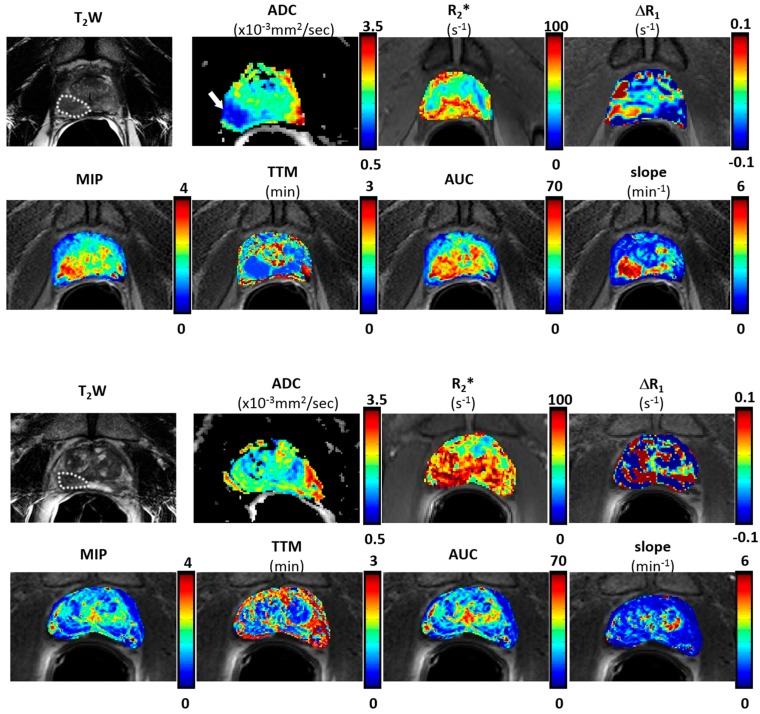
Multi-parametric MRI maps of two patients with Gleason score 8 (top panel) and 6 (bottom panel) tumors respectively. Parametric maps of prostate observed with endorectal coil at 3 T. For the patient with high Gleason score, the tumor region (white dotted lines) showed relatively higher values of R_2_* (air), maximum-intensity-projection (MIP), area-under-the-curve (AUC) and slope maps, while lower in apparent diffusion coefficient (ADC) and time-to-maximum (TTM) maps. No obvious difference was observed for the patient with a low Gleason score.

DCE images showed stable baseline (pre-contrast) signal intensity, which increased to various extents upon injecting gadolinium-based contrast. Multi-parametric maps were generated for all the patients, as presented for Patient #6 in Figure 2. The tumor exhibited lower values on ADC and TTM maps, and relatively higher values on R_2_* (both air and 100% oxygen breathing), MIP, AUC and slope compared to contralateral normal prostate tissue. Results (tumor values only) are presented for individual patients in Table 2.

**Figure 3:**
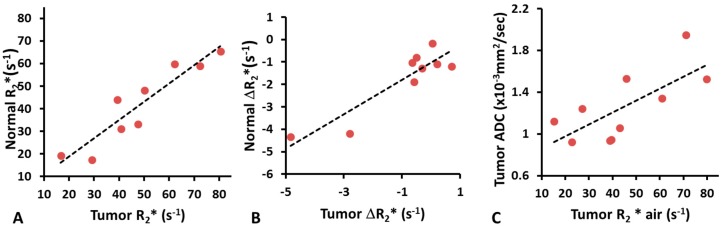
Correlations between MRI parameters. (**A**) Correlation between R_2_* of tumor and normal prostate for nine patients (*R*^2^ = 0.86; *p* < 0.001); (**B**) Correlation between ΔR_2_* of tumor and normal prostate in response to oxygen breathing challenge for nine patients (*R*^2^ = 0.82; *p* < 0.001); (**C**) Correlation between tumor ADC and R_2_* while breathing air for 10 patients (*R*^2^ = 0.53; *p* < 0.05).

Baseline R_2_* values of normal prostates and tumors were found to be correlated (*R*^2^ = 0.86; *p* < 0.001), but the tumor R_2_* (mean = 46.05 ± 20.84 s^−1^; range from 16.59 to 80.50 s^−1^, *n* = 10) was significantly higher (paired Student’s *t*-test; *p* < 0.05, *n* = 9) than that of normal prostate (mean = 41.91 ± 17.71 s^−1^; range from 17.39 to 65.30 s^−1^, *n* = 9) (Figure 3A). A wide range of baseline R_2_* values was found in normal prostate and tumor, while the R_2_* values of obturator internus or obturator externus muscles were very similar among patients (mean = 44.52 ± 4.55 s^−1^; range from 39.04 to 53.45 s^−1^). Similarly, ΔR_2_* of tumor and normal prostate upon oxygen challenge were correlated (*R*^2^ = 0.82; *p* < 0.001; Figure 3B), but ΔR_2_* was significant greater (paired Student’s *t*-test; *p* < 0.05, *n* = 9) in tumor than normal prostate (mean ΔR_2_* = −1.64 ± 1.46 s^−1^ or about −3.6%, *n* = 10; vs. −0.97 ± 1.74 s^−1^ or about −2.4%, *n* = 9). Mean R_1_ during air breathing = 0.79 ± 0.24 s^−1^ (range from 0.49 to 1.33 s^−1^) and ΔR_1_ = −0.04 ± 0.06 s^−1^: responses of individual tumors showed correlation with air R_1_ (*R*^2^ > 0.68; *p* < 0.005). Changes in R_2_* and R_1_ in tumor were not correlated (*R*^2^ < 0.01). A paired *t*-test showed significant change in R_2_* with oxygen challenge (*p* < 0.01), but not for R_1_ (*p* = 0.082). The apparent diffusion coefficient (ADC) was significantly lower (*p* < 0.001) in tumor (mean = 1.26 ± 0.33 × 10^−3^ mm^2^/s) than contralateral normal prostate (mean = 1.71 ± 0.23 × 10^−3^ mm^2^/s). ADC and R_2_* (air) showed significant correlation in tumor (*R*^2^ = 0.53; *p* < 0.05; Figure 3C). TTM was significantly lower (*p* < 0.01) and slope was significantly higher (*p* < 0.05) in tumor than contralateral normal prostate.

**Figure 4:**
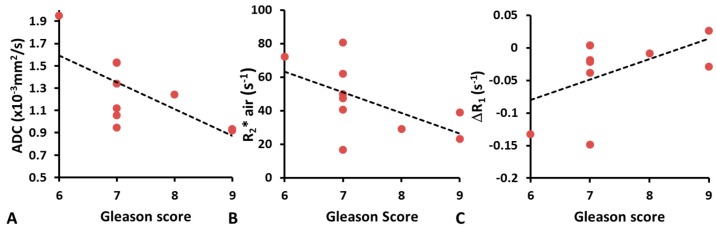
Correlations between Gleason score and multi-parametric MRI. (**A**) Correlation between ADC and Gleason score (*R*^2^ = 0.48; *p* < 0.05); (**B**) R_2_* and Gleason score while breathing air (*R*^2^ = 0.32; *p* = 0.07); (**C**) ΔR_1_ and Gleason score (*R*^2^ = 0.27; *p* = 0.08).

Moderate correlation was found between ADC and Gleason score of tumor (*R*^2^ = 0.48; *p* < 0.05; Figure 4A). Tumors with lower R_2_* values in air tended to have higher Gleason scores (*R*^2^ = 0.32; *p* = 0.07) (Figure 4B). In contrast, no significant correlations were found between the Gleason score and ΔR_1_ (Figure 4C), or any of the semi-quantitative DCE derived parameters (TTM, MIP, AUC).

**Figure 5:**
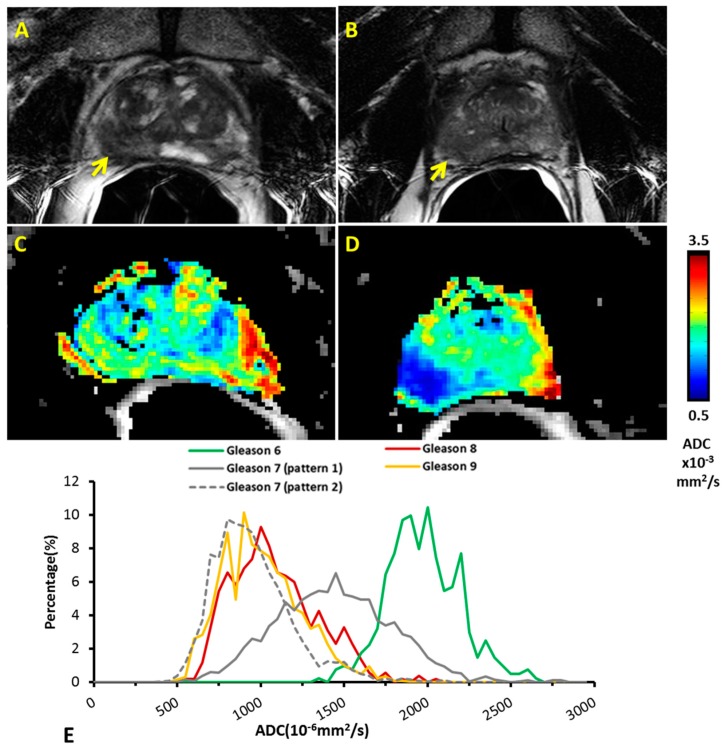
ADC maps and histogram analysis of different Gleason scores. T_2_w images showed hypointense tumor regions (yellow arrows) for both representative patients with Gleason score 6 (**A**) and 8 (**B**). They are the same patients as shown in Figure 2. ADC maps revealed little difference between tumor and normal contralateral prostate for the patient with Gleason score 6 (**C**), but clearly distinguished tumor from normal for the patient with Gleason score 8 (**D**); (**E**) Histograms show mean of ADC distributions for the groups of tumors based on voxel-by-voxel analysis. A clear separation of ADC distribution was found between the high and low Gleason score patients. Two distinct patterns were observed for those patients with intermediate Gleason score. Gleason 6: *n* = 1; Gleason 8: *n* = 1; Gleason 9: *n* = 2; Gleason 7 (pattern 1): *n* = 3; Gleason 7 (pattern 2): *n* = 3.

ADC maps showed different distribution patterns for low and high grade Gleason score tumors, as shown for two representative cases with Gleason 6 and 8 (Figure 5A–D). Tumor lesions appeared hypointense on T_2_-weighted images compared to normal prostate tissue for both patients, but only the high grade patients showed significantly decreased ADC in tumor. On a voxel-by-voxel basis distinct distributions of ADC were observed between low (Gleason 6, *n* = 1) and high (Gleason 8, *n* = 1; Gleason 9, *n* = 2) Gleason score patients (Figure 5E). Meanwhile, the intermediate Gleason scores (Gleason 7, *n* = 6) showed two different ADC distribution patterns. One pattern (*n* = 3) lay between low and high Gleason scores, and the other (*n* = 3) overlaid the high Gleason score histogram.

**Figure 6:**
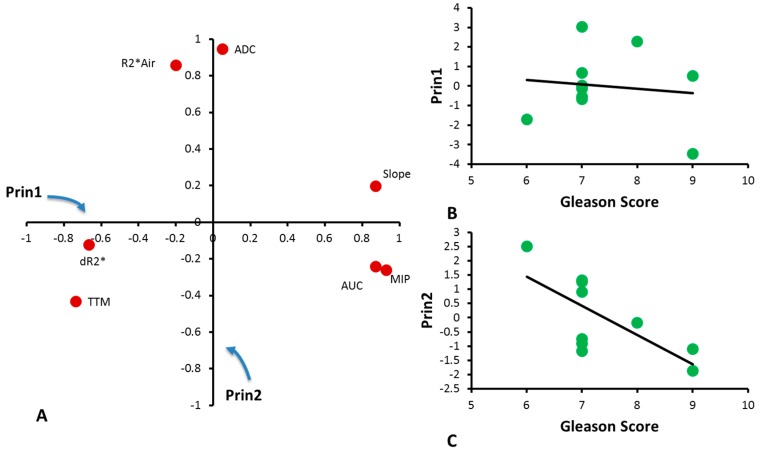
Principal Component Analysis. (**A**) Loadings of MRI parameters with Principal Components 1 and 2 describing the contribution of MRI parameters to Principal Components; (**B**) Correlation between Principal Component 1 and Gleason Score for individual tumors (*R*^2^ = 0.02; *p* = 0.72); (**C**) Correlation between Principal Component 2 and Gleason Score for individual tumors (*R*^2^ = 0.49; *p* < 0.05).

Principal Component Analysis of seven parameters in this small cohort of CaP patients identified two main Principal Components representing 49% and 29% respectively. Principal Component 1 (Prin1) is mainly attributed to ΔR_2_*, MIP, TTM, slope and AUC, while Principal Component 2 (Prin2) is mainly attributed to baseline R_2_* and ADC (Figure 6A). Prin1 and Gleason score were not correlated (Figure 6B), but a correlation between Prin2 and Gleason score was found with *R*^2^ = 0.49 and *p* < 0.05 (Figure 6C). 

## 4. Discussion

We successfully included oxygen sensitive MRI with an oxygen breathing challenge as part of a multi-parametric MRI assessment of 10 men with biopsy-proven prostate cancer as part of the planning for surgical resection.

The ability of non-invasive imaging techniques to accurately identify high Gleason CaP is critically important for the management of clinically localized CaP, including those considering active surveillance or future development of focal targeted therapies. Many MR techniques including T_2_W [33], DCE [34,35,36], DWI [37,38], and MR Spectroscopy (MRS) [35,39] have been explored for localizing and characterizing CaP and have been adopted into clinical practice. A few studies have assessed different MRI sequences simultaneously in patients: an MR protocol combining T_2_w, ADC and DCE MRI is often applied to facilitate tumor detection and grading, providing information on tumor aggressiveness [7,8,40].

In this study, we included BOLD and TOLD studies with oxygen challenge to assess the tumor response. While there were dynamic variations in R_2_* for some tumor and normal prostate tissue (Figure 1), the muscle values were quite stable during both baseline air breathing and in response to oxygen challenge. As to the average ROI measurements of individual patients, both tumor and normal prostate tissue showed a wide range of R_2_* values indicating significant individual variation, while R_2_* of reference muscle was similar among patients ensuring the variation was not scan protocol related. We found baseline tumor R_2_* was significantly higher than that of normal prostate. Significantly greater change in R_2_* was found upon oxygen breathing in tumor compared with normal prostate. No correlation was found between ΔR_2_* and Gleason score. However, ΔR_2_* showed a trend with AUC and MIP (*R*^2^ > 0.3). We also noticed that R_2_* (air and 100% oxygen) showed correlation with Gleason score. Chopra et al. [12] showed a correlation between R_2_* and hypoxic fraction < 5 Torr (HF_5_) assessed by an Eppendorf needle electrode, but did not attempt an oxygen breathing challenge. Alonzi et al. [14] used a carbogen breathing challenge (so-called carbogen lite with 2% CO_2_ to mitigate patient stress) and reported a reduction in R_2_* in tumors for nine of 14 patients with a mean reduction in R_2_* of 3.52 s^−1^ or 21.6% (*p* = 0.0005) at 1.5 T. We observed a mean reduction ΔR_2_* = 1.64 s^−1^ for the 10 tumors, which was significant for the group based on paired *t*-test (*p* < 0.01). Diergarten et al. [16] used a conventional carbogen challenge (5% CO_2_) to examine 29 patients with biopsy proven cancer followed by radical prostatectomy and reported BOLD response to be lower than normal contralateral prostate at 1.5 T. These previous studies did not attempt to correlate BOLD response with Gleason score or other MRI parameters. The ability to induce changes in tumor R_2_* using oxygen rather than carbogen breathing suggests easier implementation into human studies, since carbogen is recognized as frequently causing respiratory distress. However, highly variable response was observed, which was transient in many cases. Such behavior has also been reported in pre-clinical studies of human PC3 xenografts in mice and large Dunning prostate R3327-AT1 tumors in rats [14,18]. Given the small ΔR_2_*, it is not surprising that ΔR_1_ was not significant, since the TOLD response is generally reported to be much smaller than BOLD [18]. The small changes are commensurate with hypoxic tumors, which are not readily amenable to modulation [24], though this has not been confirmed here. BOLD MRI with an oxygen breathing challenge has previously been applied to patients with cancer of the cervix at 3 T [41]. The R_2_* values tended to be somewhat smaller in cervical tumors (mean 23.5 s^−1^) with a mean response of −1.1%, as compared with −3.6% here. It has also been reported that R_1_ of lipids may provide enhanced sensitivity to changes in pO_2_ using the so-called MOBILE approach [42], although a subsequent study at higher field found R_1_ of the water signal to be more sensitive [25]. MRI-based oxygen imaging (MOXI) [43] has been reported to provide quantitative tumor oxygenation measurements in preclinical settings and it will be interesting to apply such techniques to cancer patients in due course.

Diffusion weighted MR imaging is a noninvasive technique that explores the Brownian motion of water in tissue. Recent studies showed that ADC was useful in detecting high grade lesions that might be missed by standard biopsies [37]. We found moderate correlation between ADC and Gleason score (*R*^2^ = 0.48; *p* < 0.05) in line with previous reports [7,37,38,44]. The different ADC between high and low grade prostate tumor likely reflects the increase in cellularity as tumors progress [45]. We noticed that patients with Gleason score 7 had two distinct histogram patterns. Three patients showed ADC distribution similar to high grade classification. A wide range of ADC values for Gleason score 7 have been reported previously, potentially associated with a wide range of cellularity and biological behaviors for intermediate Gleason score 7 [40,46,47,48]. Since our patients underwent prostatectomy soon after MRI, we were not able to relate any of the MRI results with tumor progression. This could be explored in patients undergoing radiation and/or chemotherapy.

Principal Component Analysis is a statistical technique widely used for data reduction. It has previously been used in DCE analysis of CaP to recognize different enhancement patterns without the need for kinetic modeling [49]. Principal Component Analysis does not appear to have been applied to multi-parametric MRI previously. The main Principal Components identified by Principal Component Analysis represent the blood perfusion-related variables (Prin1, which is mainly attributed to ΔR_2_*, MIP, TTM, slope and AUC) and tissue composition and cellularity-related variables (Prin2, which is mainly attributed to baseline R_2_* and ADC). Correlation of the Gleason score with Prin2, but not Prin1, is reasonable, considering that the Gleason score is based on the cellular composition of the specimens. Further investigation of Principal Component Analysis with respect to histology could enhance the understanding of the Principal Components, and results will need to be validated in a larger study. We examined feasibility of data reduction based on Principal Component Analysis, but another approach is to evaluate multiparametric characteristics of tumor regions, based on cluster analysis or using one parameter to identify regions of response and apply a mask to a second parameter. Recent work reported that DCE could be used to identify well-perfused areas from hypoxic regions, and assist in correlating TOLD response with histological assessments in some tumors [50].

This study presents preliminary results of BOLD and TOLD responses to oxygen breathing in a small cohort of CaP patients. The number of patients and especially number of patients with low and high Gleason score was small, limiting the strength of the conclusions. A larger study should allow further risk stratification, including criteria such as tumor bulk, and PSA values and dynamics, as per the National Comprehensive Cancer Network (NCCN) guidelines. Correlation with the MRI-derived Prostate Imaging Reporting and Data System (PI-RADS) scores [4,51] and the Prostate Diagnostic Imaging Consensus Meeting panel proposed five-point Likert scaling [2,3] will also be pertinent, but the goal here was proof of principle to demonstrate the feasibility of including oxygen-enhanced MRI. In particular, a TOLD response has not been reported in human CaP patients previously. Rigorous immunohistological correlates could also be valuable, and the recent development of 3D printed customized molds will ensure better registration of imaging and histology. Another limitation was the use of only three *b*-values (0, 500 and 1000), due to time constraints. This limited the accuracy of ADC calculation and prevented further analysis with more sophisticated algorithms, such as the Intravoxel Incoherent Motion (IVIM) modeling.

## 5. Conclusions

Tumor ADC, R_2_*, ΔR_2_*, TTM and slope were found to be significantly different from normal prostate, and ADC and R_2_* showed general inverse trends compared to the Gleason score. Incorporating an oxygen breathing challenge into a patient imaging session was feasible, confirming the potential for further investigation. A multi-parametric approach promises further insights into pathophysiological information of the tumor microenvironment. 

## Figures and Tables

**Table 1:** Pathological tumor characteristics.

Patient	Gleason Score		1° Grade	2° Grade	3° Grade	pStage	Size (Longest Diameter by Pathology) in mm
1	7	Lesion 1	4 (50%)	3 (50%)		T3aN0	20
		Lesion 2	4 (80%)	3 (20%)			
2	7		4 (70%)	3 (30%)		T3aN0	17
3	9		4 (85%)	5 (10%)	3 (10%)	T3bN0	22
4	6		3 (100%)	3		T2aN0	9
5	7		4 (70%)	3 (30%)		T3aN0	16
6	8		4 (90%)	4		T3aN0	17
7	7		4 (55%)	3 (45%)		T2cN0	21
8	7		4 (70%)	3 (30%)		T3bN0	20
9	7	Lesion 1	4 (50%)	3 (45%)	5 (5%)	T3bN1	35
		Lesion 2	3 (100%)	3			
10	9	Lesion 1	4 (70%)	5 (20%)	3 (10%)	T3bN0	28
		Lesion 2	3 (100%)	3			

**Table 2:** Multi-parametric MRI for ten patients (tumor values).

Patient	R_2_* air (s^−1^)	R_2_* Oxygen (s^−1^)	ΔR_2_* (s^−1^)	R_1_ Air (s^−1^)	R_1_ Oxygen (s^−1^)	ΔR_1_ (s^−1^)	ADC (× 10^−3^ mm^2^/s)	MIP	TTM (min)	Slope (min^−1^)	AUC
1	62.1	61.0	−1.1	1.33	1.18	−0.15	1.34	1.48	0.55	2.70	31.84
2	50.0	45.8	−4.2	0.83	0.83	0.00	1.53	2.35	0.61	3.94	53.86
3	23.1	22.7	−0.4	0.49	0.51	0.03	0.92	2.19	1.22	1.81	51.39
4	72.2	71.0	−1.2	1.00	0.87	−0.13	1.95	1.13	1.04	1.19	26.63
5	80.5	79.7	−0.8	0.74	0.72	−0.02	1.53	1.73	1.28	1.66	40.91
6	29.0	27.1	−1.9	0.87	0.86	−0.01	1.24	2.34	0.55	4.25	44.32
7	47.4	43.1	−4.3	0.66	0.63	−0.04	1.06	2.07	1.47	1.44	46.28
8	16.6	15.3	−1.3	0.54	0.52	−0.02	1.12	1.59	1.16	1.44	37.51
9	40.6	39.5	−1.1	0.70	0.71	0.00	0.95	1.87	1.05	1.82	42.46
10	39.0	38.9	−0.1	0.77	0.74	−0.03	0.94	0.97	2.02	0.55	19.40
Mean ± SD	46.1 ± 20.8	44.4 ± 20.8	−1.6 ± 1.5	0.79 ± 0.24	0.76 ± 0.20	− 0.04 ± 0.06	1.26 ± 0.33	1.77 ± 0.48	1.10 ± 0.46	2.08 ± 1.19	39.46 ± 10.85

ADC, Apparent Diffusion Coefficient; TTM, Time-To-Maximum; AUC, Area-Under-the-Curve.

## References

[1] Pound C.R., Partin A.W., Eisenberger M.A., Chan D.W., Pearson J.D., Walsh P.C. (1999). Natural history of progression after PSA elevation following radical prostatectomy. JAMA.

[2] Costa D.N., Lotan Y., Rofsky N.M., Roehrborn C., Liu A., Hornberger B., Xi Y., Francis F., Pedrosa I. (2016). Assessment of prospectively assigned likert scores for targeted magnetic resonance imaging-transrectal ultrasound fusion biopsies in patients with suspected prostate cancer. J. Urol..

[3] Harada T., Abe T., Kato F., Matsumoto R., Fujita H., Murai S., Miyajima N., Tsuchiya K., Maruyama S., Kudo K. (2015). Five-point Likert scaling on MRI predicts clinically significant prostate carcinoma. BMC Urol..

[4] Park S.Y., Jung D.C., Oh Y.T., Cho N.H., Choi Y.D., Rha K.H., Hong S.J., Han K. (2016). Prostate Cancer: PI-RADS version 2 helps preoperatively predict clinically significant cancers. Radiology.

[5] Rastinehad A.R., Waingankar N., Turkbey B., Yaskiv O., Sonstegard A.M., Fakhoury M., Olsson C.A., Siegel D.N., Choyke P.L., Ben-Levi E. (2015). Comparison of multiparametric MRI scoring systems and the impact on cancer detection in patients undergoing MR US fusion guided prostate biopsies. PLoS ONE.

[6] Hambrock T., Somford D.M., Huisman H.J., van Oort I.M., Witjes J.A., Hulsbergen-van de Kaa C.A., Scheenen T., Barentsz J.O. (2011). Relationship between apparent diffusion coefficients at 3.0-T MR imaging and Gleason grade in peripheral zone prostate cancer. Radiology.

[7] Oto A., Yang C., Kayhan A., Tretiakova M., Antic T., Schmid-Tannwald C., Eggener S., Karczmar G.S., Stadler W.M. (2011). Diffusion-weighted and dynamic contrast-enhanced MRI of prostate cancer: Correlation of quantitative MR parameters with gleason score and tumor angiogenesis. Am. J. Roentgenol..

[8] Bloch B.N., Lenkinski R.E., Rofsky N.M. (2008). The role of magnetic resonance imaging (MRI) in prostate cancer imaging and staging at 1.5 and 3 Tesla: The Beth Israel Deaconess Medical Center (BIDMC) approach. Cancer Biomark..

[9] Alonzi R., Taylor N.J., Stirling J.J., d’Arcy J.A., Collins D.J., Saunders M.I., Hoskin P.J., Padhani A.R. (2010). Reproducibility and correlation between quantitative and semiquantitative dynamic and intrinsic susceptibility-weighted MRI parameters in the benign and malignant human prostate. J. Magn. Reson. Imaging.

[10] Tatum J.L., Kelloff G.J., Gillies R.J., Arbeit J.M., Brown J.M., Chao K.S.C., Chapman J.D., Eckelman W.C., Fyles A.W., Giaccia A.J. (2006). Hypoxia: Importance in tumor biology, noninvasive measurement by imaging, and value of its measurement in the management of cancer therapy. Int. J. Radiat. Biol..

[11] Vergis R., Corbishley C.M., Norman A.R., Bartlett J., Jhavar S., Borre M., Heeboll S., Horwich A., Huddart R., Khoo V. (2008). Intrinsic markers of tumour hypoxia and angiogenesis in localised prostate cancer and outcome of radical treatment: A retrospective analysis of two randomised radiotherapy trials and one surgical cohort study. Lancet Oncol..

[12] Chopra S., Foltz W.D., Milosevic M.F., Toi A., Bristow R.G., Menard C., Haider M.A. (2009). Comparing oxygen-sensitive MRI (BOLD R_2_*) with oxygen electrode measurements: A pilot study in men with prostate cancer. Int. J. Radiat. Biol..

[13] Turaka A., Buyyounouski M.K., Hanlon A.L., Horwitz E.M., Greenberg R.E., Movsas B. (2012). Hypoxic prostate/muscle PO_2_ ratio predicts for outcome in patients with localized prostate cancer: Long-term results. Int. J. Radiat. Oncol. Biol. Phys..

[14] Alonzi R., Padhani A.R., Maxwell R.J., Taylor N.J., Stirling J.J., Wilson J.I., d’Arcy J.A., Collins D.J., Saunders M.I., Hoskin P.J. (2009). Carbogen breathing increases prostate cancer oxygenation: A translational MRI study in murine xenografts and humans. Br. J. Cancer.

[15] Hoskin P.J., Carnell D.M., Taylor N.J., Smith R.E., Stirling J.J., Daley F.M., Saunders M.I., Bentzen S.M., Collins D.J., d’Arcy J.A. (2007). Hypoxia in prostate cancer: Correlation of BOLD-MRI with pimonidazole immunohistochemistry—Initial observations. Int. J. Radiat. Oncol. Biol. Phys..

[16] Diergarten T., Martirosian P., Kottke R., Vogel U., Stenzl A., Claussen C.D., Schlemmer H.P. (2005). Functional characterization of prostate cancer by integrated magnetic resonance imaging and oxygenation changes during carbogen breathing. Invest. Radiol..

[17] Burrell J.S., Walker-Samuel S., Baker L.C.J., Boult J.K.R., Jamin Y., Halliday J., Waterton J.C., Robinson S.P. (2013). Exploring ΔR_2_* and ΔR_1_ as imaging biomarkers of tumor oxygenation. J. Magn. Reson. Imaging.

[18] Zhao D., Pacheco-Torres J., Hallac R.R., White D., Peschke P., Cerdán S., Mason R.P. (2015). Dynamic oxygen challenge evaluated by NMR T_1_ and T_2_*—Insights into tumor oxygenation. NMR Biomed..

[19] Jiang L., Zhao D., Constantinescu A., Mason R.P. (2004). Comparison of BOLD contrast and Gd-DTPA Dynamic Contrast Enhanced imaging in rat prostate tumor. Magn. Reson. Med..

[20] Taylor N.J., Baddeley H., Goodchild K.A., Powell M.E.B., Thoumine M., Culver L.A., Stirling J.J., Saunders M.I., Hoskin P.J., Phillips H. (2001). BOLD MRI of human tumor oxygenation during carbogen breathing. J. Magn. Reson. Imaging.

[21] Matsumoto K., Bernardo M., Subramanian S., Choyke P., Mitchell J.B., Krishna M.C., Lizak M.J. (2006). MR assessment of changes of tumor in response to hyperbaric oxygen treatment. Magn. Reson. Med..

[22] O'Connor J.P.B., Naish J.H., Parker G.J.M., Waterton J.C., Watson Y., Jayson G.C., Buonaccorsi G.A., Cheung S., Buckley D.L., McGrath D.M. (2009). Preliminary study of oxygen-enhanced longitudinal relaxation in MRI: A potential novel biomarker of oxygenation changes in solid tumors. Int. J. Radiat. Oncol. Biol. Phys..

[23] Beeman S.C., Shui Y.-B., Perez-Torres C.J., Engelbach J.A., Ackerman J.J.H., Garbow J.R. (2016). O_2_-sensitive MRI distinguishes brain tumor versus radiation necrosis in murine models. Magn. Reson. Med..

[24] Hallac R.R., Zhou H., Pidikiti R., Song K., Stojadinovic S., Zhao D., Solberg T., Peschke P., Mason R.P. (2014). Correlations of noninvasive BOLD and TOLD MRI with pO_2_ and relevance to tumor radiation response. Magn. Reson. Med..

[25] Cao-Pham T.T., Tran L.B.A., Colliez F., Joudiou N., El Bachiri S., Gregoire V., Leveque P., Gallez B., Jordan B.F. (2016). Monitoring tumor response to carbogen breathing by oxygen-sensitive magnetic resonance parameters to predict the outcome of radiation therapy: A preclinical study. Int. J. Radiat. Oncol. Biol. Phys..

[26] Rodrigues L.M., Howe F.A., Griffiths J.R., Robinson S.P. (2004). Tumor R_2_* is a prognostic indicator of acute radiotherapeutic response in rodent tumors. J. Magn. Reson. Imaging.

[27] Belfatto A., White D.A., Mason R.P., Zhang Z., Stojadinovic S., Baroni G., Cerveri P. (2016). Tumor radio-sensitivity assessment by means of volume data and magnetic resonance indices measured on prostate tumor bearing rats. Med. Phys..

[28] White D.A., Zhang Z., Li L., Gerberich J., Stojadinovic S., Peschke P., Mason R.P. (2016). Developing oxygen-enhanced magnetic resonance imaging as a prognostic biomarker of radiation response. Cancer Lett..

[29] Hallac R.R., Zhou H., Pidikiti R., Song K., Solberg T., Kodibagkar V.D., Peschke P., Mason R.P. (2016). A role for dynamic contrast-enhanced magnetic resonance imaging in predicting tumour radiation response. Br. J. Cancer.

[30] Remmele S., Sprinkart A.M., Muller A., Traber F., von Lehe M., Gieseke J., Flacke S., Willinek W.A., Schild H.H., Senegas J. (2013). Dynamic and simultaneous MR measurement of R_1_ and R_2_* changes during respiratory challenges for the assessment of blood and tissue oxygenation. Magn. Reson. Med..

[31] Ahmed H.U. (2009). The Index Lesion and the Origin of Prostate Cancer. New. Engl. J. Med..

[32] Van der Kwast T.H., Amin M.B., Billis A., Epstein J.I., Griffiths D., Humphrey P.A., Montironi R., Wheeler T.M., Srigley J.R., Egevad L. (2011). International society of urological pathology (ISUP) consensus conference on handling and staging of radical prostatectomy specimens. working group 2: T2 substaging and prostate cancer volume. Mod. Pathol..

[33] Viswanath S.E., Bloch N.B., Chappelow J.C., Toth R., Rofsky N.M., Genega E.M., Lenkinski R.E., Madabhushi A. (2012). Central gland and peripheral zone prostate tumors have significantly different quantitative imaging signatures on 3 tesla endorectal, in vivo T2-weighted MR imagery. J. Magn. Reson. Imaging.

[34] McMahon C.J., Bloch B.N., Lenkinski R.E., Rofsky N.M. (2009). Dynamic contrast-enhanced MR imaging in the evaluation of patients with prostate cancer. Magn. Reson. Imaging Clin. North Am..

[35] Fütterer J.J., Heijmink S.W.T.P.J., Scheenen T.W.J., Veltman J., Huisman H.J., Vos P., Hulsbergen-Van De Kaa C.A., Witjes J.A., Krabbe P.F.M., Heerschap A. (2006). Prostate cancer localization with dynamic contrast-enhanced mr imaging and proton MR spectroscopic imaging. Radiology.

[36] Engelbrecht M.R., Huisman H.J., Laheij R.J.F., Jager G.J., van Leenders G.J.L.H., Hulsbergen-Van De Kaa C.A., de la Rosette J.J.M.C.H., Blickman J.G., Barentsz J.O. (2003). Discrimination of prostate cancer from normal peripheral zone and central gland tissue by using dynamic contrast-enhanced MR Imaging. Radiology.

[37] Bittencourt L., Barentsz J., de Miranda L., Gasparetto E. (2012). Prostate MRI: Diffusion-weighted imaging at 1.5T correlates better with prostatectomy Gleason grades than TRUS-guided biopsies in peripheral zone tumours. Eur. Radiol..

[38] Vargas H.A., Akin O., Franiel T., Mazaheri Y., Zheng J., Moskowitz C., Udo K., Eastham J., Hricak H. (2011). Diffusion-weighted endorectal MR imaging at 3 T for prostate cancer: Tumor detection and assessment of aggressiveness. Radiology.

[39] Rajesh A., Coakley F.V. (2004). MR imaging and MR spectroscopic imaging of prostate cancer. Magn. Reson. Imaging Clin. North Am..

[40] Langer D.L., van der Kwast T.H., Evans A.J., Trachtenberg J., Wilson B.C., Haider M.A. (2009). Prostate Cancer Detection with multi-parametric MRI: Logistic regression analysis of quantitative T2, diffusion-weighted imaging, and dynamic contrast-enhanced MRI. J. Magn. Reson. Imaging.

[41] Hallac R.R., Ding Y., Yuan Q., McColl R.W., Lea J., Sims R.D., Weatherall P.T., Mason R.P. (2012). Oxygenation in cervical cancer and normal uterine cervix assessed using blood oxygenation level-dependent (BOLD) MRI at 3 T. NMR Biomed..

[42] Safronova M.M., Colliez F., Magat J., Joudiou N., Jordan B.F., Raftopoulos C., Gallez B., Duprez T. (2016). Mapping of global R1 and R2* values versus lipids R1 values as potential markers of hypoxia in human glial tumors: A feasibility study. Magn. Reson. Imaging.

[43] Zhang Z., Hallac R.R., Peschke P., Mason R.P. (2014). A noninvasive tumor oxygenation imaging strategy using magnetic resonance imaging of endogenous blood and tissue water. Magn. Reson. Med..

[44] Simpkin C.J., Morgan V.A., Giles S.L., Riches S.F., Parker C., deSouza N.M. (2013). Relationship between T-2 relaxation and apparent diffusion coefficient in malignant and non-malignant prostate regions and the effect of peripheral zone fractional volume. Br. J. Radiol..

[45] Gibbs P., Liney G.P., Pickles M.D., Zelhof B., Rodrigues G., Turnbull L.W. (2009). Correlation of ADC and T2 measurements with cell density in prostate cancer at 3.0 Tesla. Investig. Radiol..

[46] Langer D.L., van der Kwast T.H., Evans A.J., Sun L., Yaffe M.J., Trachtenberg J., Haider M.A. (2008). Intermixed normal tissue within prostate cancer: Effect on mr imaging measurements of apparent diffusion coefficient and T2—Sparse versus dense cancers. Radiology.

[47] Chan T.Y., Partin A.W., Walsh P.C., Epstein J.I. (2000). Prognostic significance of Gleason score 3+4 versus Gleason score 4+3 tumor at radical prostatectomy. Urology.

[48] Sakr W.A., Tefilli M.V., Grignon D.J., Banerjee M., Dey J., Gheiler E.L., Tiguert R., Powell I.J., Wood D.P. (2000). Gleason score 7 prostate cancer: A heterogeneous entity? Correlation with pathologic parameters and disease-free survival. Urology.

[49] Eyal E., Bloch B.N., Rofsky N.M., Furman-Haran E., Genega E.M., Lenkinski R.E., Degani H. (2010). Principal component analysis of dynamic contrast enhanced MRI in human prostate cancer. Invest. Radiol..

[50] O‘Connor J.P.B., Boult J.K.R., Jamin Y., Babur M., Finegan K.G., Williams K.J., Little R.A., Jackson A., Parker G.J.M., Reynolds A.R. (2016). Oxygen-enhanced MRI accurately identifies, quantifies, and maps tumor hypoxia in preclinical cancer models. Cancer Res..

[51] Weinreb J.C., Barentsz J.O., Choyke P.L., Cornud F., Haider M.A., Macura K.J., Margolis D., Schnall M.D., Shtern F., Tempany C.M. (2016). PI-RADS prostate imaging—Reporting and data system: 2015, version 2. Eur. Urol..

